# Understanding the role of the gut microbiome in solid tumor responses to immune checkpoint inhibitors for personalized therapeutic strategies: a review

**DOI:** 10.3389/fimmu.2024.1512683

**Published:** 2025-01-07

**Authors:** Mi Young Lim, Seungpyo Hong, Young-Do Nam

**Affiliations:** ^1^ Personalized Diet Research Group, Korea Food Research Institute, Wanju-gun, Jeollabuk-do, Republic of Korea; ^2^ Department of Molecular Biology, Jeonbuk National University, Jeonju-si, Jeollabuk-do, Republic of Korea

**Keywords:** gut microbiome, immunotherapy, immune checkpoint inhibitor, solid cancer, response, prediction, intervention

## Abstract

Immunotherapy, especially immune checkpoint inhibitor (ICI) therapy, has yielded remarkable outcomes for some patients with solid cancers, but others do not respond to these treatments. Recent research has identified the gut microbiota as a key modulator of immune responses, suggesting that its composition is closely linked to responses to ICI therapy in cancer treatment. As a result, the gut microbiome is gaining attention as a potential biomarker for predicting individual responses to ICI therapy and as a target for enhancing treatment efficacy. In this review, we discuss key findings from human observational studies assessing the effect of antibiotic use prior to ICI therapy on outcomes and identifying specific gut bacteria associated with favorable and unfavorable responses. Moreover, we review studies investigating the possibility of patient outcome prediction using machine learning models based on gut microbiome data before starting ICI therapy and clinical trials exploring whether gut microbiota modulation, for example via fecal microbiota transplantation or live biotherapeutic products, can improve results of ICI therapy in patients with cancer. We also briefly discuss the mechanisms through which the gut microbial-derived products influence immunotherapy effectiveness. Further research is necessary to fully understand the complex interactions between the host, gut microbiota, and immunotherapy and to develop personalized strategies that optimize responses to ICI therapy.

## 1 Introduction

Immunotherapy harnesses the body’s immune system to target and eliminate cancer cells (1). The various forms of immunotherapy include immune checkpoint inhibitors (ICIs)—such as programmed cell death protein 1 (PD-1), programmed cell death ligand 1 (PD-L1), and cytotoxic T-lymphocyte–associated antigen 4 (CTLA-4) inhibitors—, adoptive cell therapies—such as chimeric antigen receptor T-cell therapy, tumor-infiltrating lymphocyte therapy, and natural killer (NK) cell therapy—, cancer vaccines, and cytokine therapies (2). These therapies work by either enhancing the immune system’s ability to detect and destroy cancer cells or infusing cancer-fighting immune cells that effectively target cancer cells (3).

Among various types of cancer immunotherapy, ICI therapy has emerged as a promising therapeutic approach for a variety of solid tumors, including for non-small cell lung cancer (NSCLC) and melanoma (4). ICIs are monoclonal antibodies that can target and block immune checkpoints, such as PD-1 and CTLA-4 on T cells or PD-L1 on tumor cells, thus inhibiting tumor immune escape and enhancing the antitumor function of T cells (5). ICIs offer clear advantages such as high accuracy, response durability, wide adaptability, and low toxicity (2). However, the effectiveness of ICI therapy can vary greatly between individuals. For instance, in patients with NSCLC with high PD-L1 expression treated with pembrolizumab (a PD-1 inhibitor), the five-year overall survival (OS) rate is approximately 25–30% (6). A recent study estimated that only 19.60% of US patients with cancer responded to ICI treatment in 2023. This high variability in ICI response has been linked to several factors, including tumor mutational burden, PD-L1 expression, immune infiltration, altered interferon (IFN)-γ signaling, and epigenetic modifications (7, 8). Nevertheless, the factors determining the efficacy of ICI therapy remain poorly understood, posing considerable challenges for improving response rates and developing effective, patient-specific ICI treatment strategies.

Recent studies have shown that the gut microbiota plays a significant role in shaping drug responses by either modifying drug structures or affecting host physiological functions (9–11). Moreover, the gut microbiota can modulate local and systemic immune responses via microbial metabolites or components (12). For example, gut microbiota can affect T-cell expression profiles (e.g., regulatory T cells (Tregs) and CD8+ T cells) and regulate the differentiation of monocytes into immunogenic dendritic cells (DCs) (13, 14). Furthermore, short-chain fatty acid (SCFA) butyrate produced by gut microbiota can enhance CD8+ T-cell effector function and promote the transition of activated CD8+ T cells to T memory cells (15, 16). These immunomodulatory capacities of the gut microbiota suggest its possible influence on the efficacy of ICI therapy. In 2015, Vétizou et al. showed that antibiotic-treated or germ-free mice with tumors exhibited poor responses to CTLA-4 blockade, and the supplementation of specific *Bacteroides* spp. restored treatment efficacy by activating T-helper (Th) 1 cells in tumor-draining lymph nodes and promoting the maturation of intratumoral DCs (17). Importantly, three studies in 2018 have revealed a strong link between the gut microbiota and outcomes of ICI therapy in human (18–20), positioning it as a potential biomarker for predicting immunotherapy response and a modifiable target for enhancing treatment efficacy.

**Figure 1:**
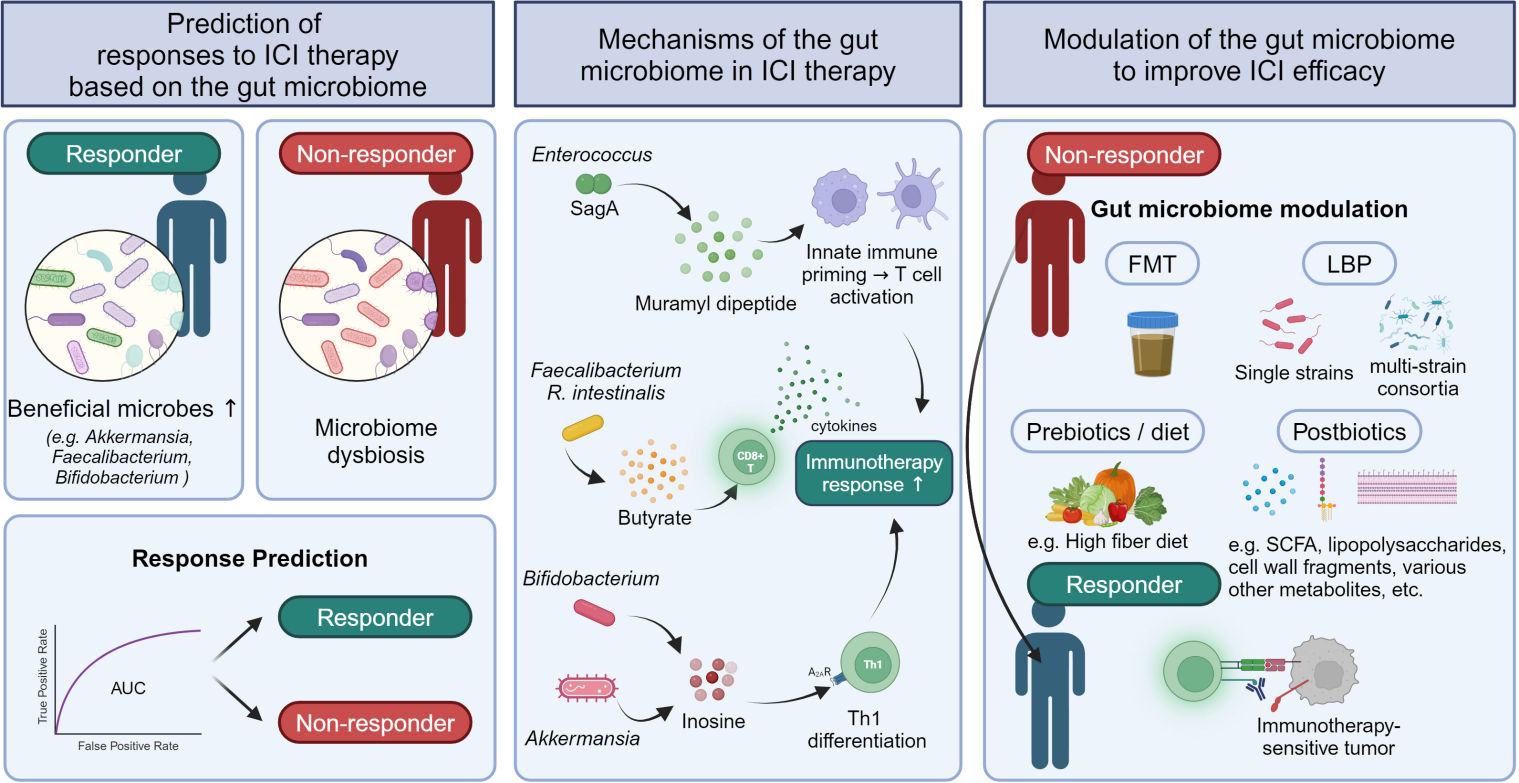
Strategies to predict ICI responses and improve ICI efficacy based on the influence of the gut microbiome in solid tumor responses to ICI therapy.

This review focuses on the past and current state of research on role of the gut microbiota in the responses to ICI therapy in human patients with solid tumors (**Figure 1**). We explore findings from studies investigating whether gut microbial dysbiosis due to antibiotic treatment is associated with ICI therapy outcomes in patients with solid tumors and determining which specific gut microbes are differentially abundant in responders and non-responders. We also examine research on outcome prediction using machine learning models based on gut microbiome data before starting ICI therapy, as well as clinical trials determining whether gut microbiota modulation can improve ICI treatment outcomes. In addition, we briefly outline proposed mechanisms by which the gut microbiota impacts the efficacy of ICI therapy via cell components or metabolites. As most human studies on this subject have been conducted specifically on gut bacteria, the term “gut microbiome” in this review will refer to the gut bacterial microbiome.

## 2 Impact of antibiotic treatment on efficacy of ICI therapy

Patients with cancers are often prescribed antibiotics to treat infectious diseases such as dental, respiratory tract, and urinary tract infections. Antibiotic use is necessary to control infections, but it can disrupt gut microbial homeostasis by altering microbial composition and function (21). The gut dysbiosis induced by antibiotic use can affect the host immune system (22) and has been linked to increased risks of various immunological diseases such as allergy, asthma, and rheumatoid arthritis (23–25). These findings indicate that antibiotic use in patients with cancers may influence the efficacy of ICI therapy by impairing immune responses. Since the first study reporting worsened responses to ICI therapy in antibiotic-treated mice (17), many studies have examined the impact of antibiotic use on outcomes of ICI therapy in human cohorts (**Table 1**).

**Table 1:** The relationship between antibiotic treatment and outcomes of ICI therapy.

Cancer	Type of antibiotics	Time windows of exposure to antibiotics	Duration of antibiotic therapy	Immunotherapy	Survival (antibiotics vs. no antibiotics)	Countries	References
NSCLC (n = 140), RCC (n = 67), UC (n = 42)	β-lactam+/− inhibitors, fluoroquinolones, or macrolides	2 months before or 1 month after ICI initiation (69/249, 28%)	3–151 days, or not reported	anti-PD-1/PD-L1	NSCLC+RCC+UC median PFS: 3.5 vs. 4.1 months (p = 0.017) median OS: 11.5 vs. 20.6 months (p < 0.001)	France	Routy et al., 2018 (26)
NSCLC (n = 239), RCC (n = 121)	β-lactam+/− inhibitors, quinolones, macrolides, sulfonamides, etc.	within 30 days before ICI initiation NSCLC (48/239, 20%), RCC (16/121, 13%)	≤ 7 days NSCLC (35, 73%), RCC (8, 50%) > 7 days NSCLC (13, 27%), RCC (8, 50%)	anti-PD-1/PD-L1/CTLA-4	NSCLC median PFS: 1.9 vs. 3.8 months (p = 0.03) median OS: 7.9 vs. 24.6 months (p < 0.01) RCC median PFS: 1.9 vs. 7.4 months (p < 0.01) median OS: 17.3 vs. 30.6 months (p = 0.03)	France, USA	Derosa et al., 2018 (18)
NSCLC (n = 131), Melanoma (n = 27), other solid cancers (n = 76)	β-lactam/Betalactamase inhibitors, fluoroquinolones, cephalosporins	within 60 days before ICI initiation (108/234, 46%)	NA	anti-PD-1/PD-L1/CTLA-4	median PFS: 2 vs. 4 months (p < 0.001) median OS: 5 vs. 17 months (p < 0.001)	Republic of Korea	Kim et al., 2019 (27)
RCC (n = 69)	β-lactam ± inhibitors, quinolones, or unknown	within 2 months before ICI initiation (11/69, 16%)	6–29 days	anti-PD-1	median PFS: 1.87 vs. 5.09 months (p = 0.03) median OS: 24.6 months vs. undefined (p = 0.04)	France	Derosa et al., 2020 (38)
NSCLC (n = 256)	β-lactams, fluoroquinolones, macrolides, cephalosporins, or tetracyclines	within 60 days of ICI initiation or concurrently with the first month of ICI therapy (46/256, 18%)	mean 1.12 days (for surgery) ~ mean 13.65 days (for other reasons)	anti-PD-1/PD-L1/CTLA-4	median OS: 384 vs. 506 days (p = 0.145)	USA	Nyein et al., 2022 (28)
NSCLC (n=199), Melanoma (n=222)	NA	3 months before to 1 month after ICI initiation NSCLC (61/199, 31%) Melanoma (71/222, 32%)	NA	anti-PD-1/PD-L1	NSCLC median PFS: 2.83 vs. 5.63 months (p = 0.0081) median OS: 8.6 vs. 18.5 months (p < 0.001) Melanoma median PFS: 5.77 vs. 10.2 months (p = 0.3) median OS: 19.2 vs. 35.6 months (p = 0.033)	Finland	Vihinen et al., 2023 (83)
NSCLC (n=74)	NA	2 months before and after ICI initiation (13/74, 17.6%)	NA	anti-PD-1/PD-L1	mean PFS: 4.8 vs. 6.7 months (p = 0.037) mean OS: 7.4 vs. 16.1 months (p = 0.206)	China	Luo et al., 2024 (29)

NSCLC, non-small cell lung cancer; RCC, renal cell carcinoma; UC, urothelial carcinoma; ICI, immune checkpoint inhibitors; PFS, progression-free survival; OS, overall survival.

In a large European cohort of patients with advanced NSCLC (n = 140), renal cell carcinoma (RCC) (n = 67), and urothelial carcinoma (UC) (n = 42) who received PD-1/PD-L1 inhibitors, progression-free survival (PFS) and/or OS were significantly shorter in the antibiotic-treated group within two months before or one month after the start of ICI treatment, compared to the untreated group (PFS: 3.5 months for antibiotic users vs. 4.1 months for non-users, OS: 11.5 vs. 20.6 months) (26). Similarly, detrimental effects of antibiotic use within 30 days of the first ICI administration on therapeutic efficacy were observed in patients with advanced RCC (n = 121) and NSCLC (n = 239) treated with anti-PD-L1 inhibitor (PFS: 1.9 vs. 7.4 months, OS: 17.3 vs. 30.6 months) (18). In a cohort of Korean patients with solid tumors (n = 234), including NSCLC and melanoma, antibiotic treatment within 60 days of starting ICI therapy was associated with significantly reduced PFS (2 vs. 4 months) and OS (5 vs. 17 months) (27). However, other studies have shown minimal or no significant effects of antibiotic use on outcomes of ICI therapy (28, 29). Despite these mixed findings, a meta-analysis of 23 studies on NSCLC patients reported that antibiotic use before or during ICI treatment reduced median PFS (n = 2208) by 1.2 months and median OS (n = 5560) by 6.7 months (30). Another meta-analysis of 37 studies across 13 cancer types also found that antibiotic use was significantly associated with poorer prognosis (OS and PFS) in cancer patients treated with ICIs (31).

These findings suggest that the gut microbiota plays a critical role in determining responses to ICI therapy and underscore the importance of considering a patient’s antibiotic treatment history when planning ICI treatment. More detailed studies are needed to explore the effects of different class of antibiotics, doses, treatment durations, and time of administration on the efficacy of ICI therapy in various cancer types and stages.

## 3 Gut bacterial features related with outcomes of ICI therapy

Identifying the specific microbes that play pivotal roles in influencing responses to ICI therapy is crucial for understanding their immune modulation mechanisms, predicting the efficacy of ICI therapy, and developing personalized microbiota modulation strategies to enhance outcomes of ICI therapy. In 2018, three landmark studies were published. Routy et al. found that *Akkermansia muciniphila*, *Enterococcus faecium*, and *Alistipes indistinctus* were significantly enriched in responders compared to non-responders among patients with NSCLC and RCC treated with PD-1 inhibitors (n = 100) (26). Matson et al. reported enrichment of *Bifidobacterium longum*, *Collinsella aerofaciens*, and *E. faecium* in responders (n = 42), while Gopalakrishnan et al. found enrichment of *Faecalibacterium* in responders (n = 43) in melanoma patients treated with PD-1/CTLA-4 inhibitors (19, 20).

Since these initial studies, many others have reported distinct gut microbial features associated with responses to ICI therapy (**Table 2**). In patients with advanced NSCLC treated with anti-PD-1 blockade (n = 25), responders had a higher abundance of *Alistipes putredinis*, *Prevotella copri*, and *B. longum*, while non-responders had more *Ruminococcus* (32). A large cohort study of NSCLC patients receiving ICIs (n = 338) found that baseline *A. muciniphila* presence was associated with higher objective response rates (ORR) and OS (33). Another study of NSCLC patients (n = 65) undergoing ICI therapy reported significant enrichment of *Ruminococcus*, *Akkermansia*, and *Faecalibacterium* in responders (34). In addition to NSCLC, several studies have been conducted on melanoma. In melanoma patients receiving ICI therapy (n = 27), prolonged PFS was linked to higher abundances of *Faecalibacterium prausnitzii*, *Coprococcus eutactus*, and *Prevotella stercorea*, while shorter PFS was associated with higher levels of *Bacteroides ovatus*, *B. dorei*, *B. massiliensis*, *Ruminococcus gnavus*, and *Blautia producta* (35). Another study of advanced melanoma patients treated with combined CTLA-4 and PD-1 blockade (n = 40) found that responders had enriched levels of *Bacteroides stercoris*, *Parabacteroides distasonis*, and *Fournierella massiliensis* (36). In RCC (n = 31), *Bifidobacterium adolescentis*, *Barnesiella intestinihominis*, *Odoribacter splanchnicus*, and *Bacteroides eggerthii* were enriched in responders (37). Another RCC study (n = 58) showed that responders to PD-1 inhibitors had higher levels of *A. muciniphila*, *Bacteroides salyersiae*, and *Eubacterium siraeum* (38). In hepatocellular carcinoma (HCC), an increase in *Faecalibacterium* was observed in patients who responded to ICI therapy (39, 40).

**Table 2:** Gut bacterial features associated with responses to ICI therapy.

Cancer	Immunotherapy	Sample size	Responders-associated with bacteria	Non-responders-associated with bacteria	Sequencing methods	Countries	Reference
Melanoma	anti-PD-1/CTLA-4	42	*Enterococcus faecium, Collinsella aerofaciens, Bifidobacterium adolescentis, Klebsiella pneumoniae, Veillonella parvula, Parabacteroides merdae, Lactobacillus* sp.*, Bifidobacterium longum*	*Ruminococcus obeum, Roseburia intestinalis*	16S V4/MGS/qPCR	USA	Matson et al., 2018 (20)
NSCLC, RCC	anti-PD-1	100	*Akkermansia muciniphila, Enterococcus faecium, Alistipes indistinctus*	*Parabacteroides distasonis, Bacteroides nordii, Blautia, Bacteroides clarus*	MGS	France	Routy et al., 2018 (26)
Melanoma	anti-PD-1	43	*Faecalibacterium*	*Bacteroides thetaiotaomicron, Escherichia coli, Anaerotruncus colihominis*	16S V4/MGS	USA	Gopalakrishnan et al., 2018 (19)
Melanoma	anti-PD-1/CTLA-4/PD-1+CTLA-4	27	*Faecalibacterium prausnitzii, Coprococcus eutactus, Prevotella stercorea, Streptococcus sanguinis, Streptococcus anginosus, Lachnospiraceae bacterium_3_1_46FAA*	*Bacteroides ovatus, Bacteroides dorei, Bacteroides massiliensis, Ruminococcus gnavus, Blautia producta*	16S V4/MGS	USA	Peters et al., 2019 (35)
NSCLC	anti-PD-1	25	*Alistipes putredinis, Prevotella copri, Bifidobacterium longum, Lachnobacterium, Lachnospiraceae, Shigella*	*Ruminococcus_unclassified*	16S V3-V4	China	Jin et al., 2019 (32)
RCC	anti-PD-1/PD-1+CTLA-4	31	*Bifidobacterium adolescentis, Barnesiella intestinihominis, Odoribacter splanchnicus, Bacteroides eggerthii*	*Bacteroides ovatus, Eggerthella lenta, Fusicatenibacter saccharivorans, Flavonifractor plautii*	MGS	USA	Salgia et al., 2020 (37)
RCC	anti-PD-1	58	*Akkermansia muciniphila, Bacteroides salyersiae, Eubacterium siraeum*	*Erysipelotrichaceae bacterium_2_2_44A, Clostridium hathewayi, Clostridium clostridioforme*	MGS	France	Derosa et al., 2020 (38)
Melanoma (meta-analysis)	anti-PD-1/PD-L1/CTLA-4	103	Unknown Ruminococcaceae species, unknown *Faecalibacterium* species, *Ruminococcus bicirculans*, *Barnesiella intestinihominis*	*Bacteroides thetaiotaomicron*, *Adlercreutzia equolifaciens*, *Bifidobacterium dentium*, unknown *Mogibacterium*	MGS	USA	Limeta et al., 2020 (42)
Melanoma	anti-PD-1	132	*Ruminococcaceae, Faecalibacterium*		16S V4/MGS	USA	Spencer et al., 2021 (54)
HCC, BTC	anti-PD-1	65	*Lachnospiraceae bacterium-GAM79, Alistipes* sp. *Marseille-P5997, Ruminococcus callidus, Eubacterium siraeum, Gemmiger formicilis, Faecalibacterium genus*	*Veillonellaceae*	MGS	China	Mao et al., 2021 (40)
Melanoma	anti-PD-1+CTLA-4	40	*Bacteroides stercoris, Parabacteroides distasonis, Fournierella massiliensis*	*Klebsiella aerogenes, Lactobacillus rogosae*	16S V4/MGS	USA	Andrews et al., 2021 (36)
Melanoma (meta-analysis)	anti-PD-1	155	Organisms within the Firmicutes and Actinobacteria phyla	Prevotellaceae, Rikenellaceae, Porphyromonadaceae, and Bacteroidaceae families within the Bacteroidetes phylum	MGS	USA	McCulloch et al., 2022 (44)
Melanoma	anti-PD-1+CTLA-4	71	*Alistipes, Alistipes indistinctus, Alphaproteobacteria, Anaerostipes, Butyricicoccus pullicaecorum, Butyricimonas, Erysipelotrichaceae ASV, Lachnospiraceae ASV, Oscillospira ASV, RF32, Rikenellaceae, Rikenellaceae ASV, Sutterella ASV*	*Bacilli, Blautia producta ASV, Citrobacter ASV, Clostridiaceae, Clostridium rumintium ASV, Eubacterium, Faecalibacterium prausnitzii ASV, Paraprevotella ASV*, *SMB53, Turicibacter, Turicibacter ASV*	16S V4	Australia	Simpson et al., 2022 (84)
NSCLC	anti-PD-1	338	*Akkermansia muciniphila*		MGS	France, Canada	Derosa et al., 2022 (33)
HCC	anti-PD-1	41	*Prevotellaceae, Prevotella 9, Faecalibacterium*	*Veillonella, Lachnoclostridium*, Lactobacillales	16S V3-V4	Taiwan	Lee et al., 2022 (39)
NSCLC	anti-PD-1/PD-L1/PD-1+CTLA-4	65	*Ruminococcus, Akkermansia, Blautia, Faecalibacterium*	*Klebsiella, Fusobacterium, Enterococcus*	16S V1-V3	USA	Newsome et al., 2022 (34)
NSCLC	anti-PD-1	62	*Alistipes shahii*, *Barnesiella visceriola*, *Butyricimonas faecalis*, *Bacteroides* sp. *A1C1*, *Alistipes finegoldii*	*Streptococcus salivarius*, *Streptococcus vestibularis*, *Streptococcus parasanguinis*, *Bifidobacterium longum*, *Bifidobacterium adolescentis*, *Bifidobacterium breve*	MGS	Hungary	Dora et al., 2023 (47)
Mesothelioma	anti-PD-L1+VEGF	26	*Prevotella, Butyricioccus, Eubacterium ventriosum group, Biophilia*	*Erysipeloclostridium*	16S V3-V4	UK	Zhang et al., 2024 (85)
HCC	anti-PD-1/PD-L1	188	*Phascolarctobacterium faecium, Candidatus Avimonas narfia*	*Actinomyces sp ICM47, Senegalimassilia anaerobia, Faecalibacillus faecis*	MGS	China	Zhu et al., 2024 (86)
GI cancer	anti-PD-1/PD-L1	77	*Bacteroides caccae, Porphyromonadaceae, Parabacteroides, Acidaminococcaceae, Alistipes finegoldii, Paraprevotella clara, Bacteroides massiliensis, Alistipes putredinis*	*Veillonella parvula, Veillonella atypica, Peptostreptococcaceae, Streptococcus thermophilus, Micrococcaceae*	MGS	China	Cheng et al., 2024 (45)
NSCLC	anti-PD-1/PD-L1	245	Species-interacting group 2 (SIG2): *Lachnospiraceae (species from the genus Blautia, Roseburia, Dorea, and Eubacterium), Oscillospiraceae (Faecalibacterium prausnitzii, Ruminococcus bicirculans, and Ruminococcus lactaris)*	Species-interacting group 1 (SIG1): *Enterocloster, Streptococcaceae, Veillonellaceae, Lactobacillaceae*	MGS	France, Canada	Derosa et al., 2024 (41)

NSCLC, non-small cell lung cancer; RCC, renal cell carcinoma; HCC, hepatocellular carcinoma; BTC, biliary tract cancer; GI, gastrointestinal; ASV, amplicon sequence variant; MGS, shotgun metagenomic sequencing.

Taken together, a wide range of individual gut microbes have been associated with outcomes of ICI therapy, with *Akkermansia* and *Faecalibacterium* frequently linked to favorable responses across different cancers. However, there is no clear consensus, making it difficult to use these microbes as universally applicable biomarkers. Recently, Derosa et al. proposed a biomarker based on the ecological topology of the gut microbiome that predicts responses to ICI therapy in various cancers (41). Using co-abundance networks, they identified two species-interacting groups (SIGs) related to PD-1 blockade response in NSCLC patients. SIG1 comprised bacteria associated with reduced OS, such as the *Enterocloster* genus and Streptococcaceae, Veillonellaceae, and Lactobacillaceae families, while SIG2 consisted of longer OS-related bacteria, such as *F. prausnitzii* and *Ruminococcus bicirculans.* The topological score (TOPOSCORE), which is a combination of the SIG1/SIG2 ratio and *A. muciniphila* abundance, was significantly associated with immunotherapy outcome in the NSCLC discovery cohort and could predict patients’ responses to ICI therapy with an area under the receiver operating characteristic curve (AUC) of 0.66. The association of the TOPOSCORE with responses to ICI was also validated in patients with NSCLC and those with genitourinary cancer, suggesting the potential of TOPOSCORE as a robust biomarker for predicting immunotherapy response (41). These findings highlight the importance of considering not only individual bacteria but also bacterial interactions when predicting ICI responses based on the gut microbiome.

## 4 Machine learning models for predicting responses to ICI therapy based on the gut microbiome

The identification of significant associations between the gut microbiome and ICI response have led researchers to investigate machine learning models to stratify patients into responders and non-responders to immunotherapy using the gut microbial profiles before starting treatment (**Table 3**).

**Table 3:** Predictive machine learning models distinguishing responders from non-responders based on the gut microbiome.

Cancer	Immunotherapy	Model algorithms	Feature	Sample size (training set)	AUC (training set)	Sample size (independent validation set)	AUC (independent validation set)	Sequencing methods	Countries	Reference
Melanoma (meta-analysis)	anti-PD-1/PD-L1/CTLA-4	RF	Differentially abundant species and pathways	103	0.604	27	0.624	MGS	USA	Limeta et al., 2020 (42)
Melanoma (meta-analysis)	anti-PD-1/CTLA-4	RF, GLM, poly-SVM	Batch-corrected taxon count data (k-means clustering, k = 150)	155		Modified LOOCV	0.54–1.00	MGS	USA	McCullochet al., 2022 (44)
NSCLC	anti-PD-1	RF	Differentially abundant species and/or pathways	37	0.74 (species), 0.63 (pathway), 0.63 (species+pathways)			MGS	Hungary	Dora et al., 2023 (47)
GI cancer (GC, CRC)	anti-PD-1/PD-L1	LightGBM	All microbial species (automated feature selection)	77 (train 8: test 2)	0.9 (test, 0.88)	31	0.79	MGS	China	Cheng et al., 2024 (45)
BTC	anti-PD-1/PD-L1	RF	All microbial species (automated feature selection)	54 (train 2: test 1)	0.897 (test, 0.722)			MGS	China	Zhu et al., 2024 (46)
NSCLC	anti-PD-1/PD-L1	RF	All microbial species	245	0.651			MGS	France, Canada	Derosa et al., 2024 (41)

NSCLC, non-small cell lung cancer; GI, gastrointestinal; GC, gastric cancer, CRC, colorectal cancer; BTC, biliary tract cancer; RF, random forest; GLM, generalized linear model; poly-SVM, polynomial support vector machine; LightGBM, light gradient-boosting machine; LOOCV, leave-one-out cross-validation; MGS, shotgun metagenomic sequencing.

Limeta et al. (42) developed a predictive model using three previously published gut microbiome datasets from 103 patients with melanoma undergoing ICI therapy (19, 20, 43). They used 49 differentially abundant species and functional pathways between responders and non-responders, including unknown Ruminococcaceae species, unknown *Faecalibacterium* species, *Bacteroides thetaiotaomicron*, and aerobic respiration, as input for training a random forest (RF) classifier. The AUC of the model on the training data was 0.604. They also evaluated the classification performance of the model on the independent validation dataset (n = 27) (35), achieving an AUC of 0.624 (42). McCullochet et al. (44) also developed machine learning models to predict the PD-1 response of patients with melanoma using 155 metagenomic datasets from a new cohort and four published independent cohorts (19, 20, 35, 43). In this study, batch-effect-corrected taxon count data were used to reduce the inter-study heterogeneity of metagenomic data, and a modified leave-one-out cross-validation method, where models were trained on the four cohorts and tested on the remaining cohort, was applied to overcome the limitation of small sample size in each cohort. Three machine learning algorithms, including RF, a generalized linear model, and a polynomial support vector machine, were used, and the resulting AUC values were between 0.54 and 1.00 (44). This study showed that machine learning models trained on gut microbiome data can consistently predict the efficacy of PD-1 therapy in patients with melanoma prior to ICI treatment.

A machine learning model for gastrointestinal (GI) cancer was developed using microbial species data from 18 patients with gastric cancer (GC) and 59 patients with colorectal cancer (CRC) undergoing anti-PD-1/PD-L1 treatment (45). The light gradient-boosting machine model showed high predictive performance (AUC of 0.90 in the training dataset), and the performance of this model was confirmed in the independent validation set (n = 31) with an AUC of 0.79. In this model, *Bacteroides caccae*, *Veillonella parvula*, *V. atypica*, and Clostridiales bacteria significantly contributed to distinguishing responders from non-responders (45). Recently, it was reported that the outcome of anti-PD-1/PD-L1 immunotherapy can be also predicted in biliary tract cancer (BTC) (46). This study used microbiome data from 77 patients with BTC and built three different RF models based on bacteria, metabolites, and a combination of bacteria and metabolites. The model trained only with six bacteria, including *Streptococcus anginosus*, *Olsenella profusa*, and *A. putredinis*, showed high predictive performance with an AUC of 0.897 for the training dataset and of 0.722 for the test dataset (46).

For NSCLC, the aforementioned study introducing the TOPOSCORE approach (41) also developed a machine learning model based on the relative abundance of all microbial species to compare the predictive performance of TOPOSCORE across 245 patients with NSCLC. The TOPOSCORE distinguished the responders and non-responders with an AUC of 0.66, while the RF model using microbiome data achieved an AUC of 0.651, indicating that these two approaches have similar performance in predicting the outcome of ICI therapy in patients with NSCLC (41). Another study including small number of patients with NSCLC (n = 37) also demonstrated RF models based on differentially abundant species and/or pathways between patients with long and short PFS. The model based on species could discriminate between long- and short-PFS patients with an AUC of 0.74 (47).

These models demonstrated moderate prediction performances, indicating the potential for gut microbial profiles to serve as markers for treatment response. Notably, the models for cancers of the GI system (GC, CRC, and BTC) exhibited higher predictive performances than those for other cancers, suggesting that ICI therapy for GI cancers may be more directly influenced by the gut microbiota. Given the complexity and variability of the gut microbiome, it is evident that further validation is necessary to enhance the clinical applicability of these predictive models.

## 5 Modulation of the gut microbiota to improve the efficacy of ICI therapy

### 5.1 Observational studies

The link between the gut microbiome and responses to ICI therapy suggests the potential to enhance the therapeutic efficacy by modulating the gut microbiota. Key strategies to do this include fecal microbiota transplantation (FMT), live biotherapeutic products (LBP: single taxa or microbial consortia), probiotics, prebiotics, postbiotics, and dietary modifications. Several observational studies have explored the relationship between probiotic or dietary fiber intake and outcomes of ICI therapy.

Interestingly, research on the effect of probiotic intake on the efficacy of solid tumor immunotherapy is underway in Japan with a particular focus on the *Clostridium butyricum* MIYAIRI 588 strain (CBM588). CBM588 is an anaerobic, butyrate-producing, Gram-positive bacteria widely used for the treatment of diarrhea in Japan (48). Recent studies have revealed that CBM588-based probiotics can promote the growth of *Bifidobacterium* and *Lactobacillus*, enhance the intestinal barriers, and modulate immune function and inflammation (49, 50). A retrospective study investigated whether the intake of strain CBM588 before and/or during ICI therapy affected survival and response to ICI in 118 Japanese patients with advanced NSCLC. Probiotic intake significantly prolonged PFS (250 days for probiotic users vs. 101 days for non-users) and OS (not reached vs. 361 days). The use of strain CBM588 was also associated with improved ICI efficacy in a subgroup analysis of patients who received antibiotic therapy prior to treatment initiation (51). Another study of 294 Japanese patients with NSCLC found that PFS was significantly longer in those taking probiotics, including *Bifidobacterium* (BIOFERMIN and LAC-B), CBM588 (MIYA-BM), and antibiotic-resistant lactic acid bacteria, at the time of anti-PD-1 therapy initiation compared to those not taking probiotics (52). Similarly, among 482 Japanese NSCLC patients receiving ICI monotherapy, those who took probiotics before or during ICI therapy had longer PFS (7.9 months for probiotic users vs. 2.9 months for non-users) and better OS (not reached vs. 13.1 months). In this study, the patients who took probiotics were further divided into two groups according to the probiotic types that they took, CBM588 vs non-spore-forming bacteria only, and no significant differences in PFS and OS depending on the probiotic types were observed (53). These studies indicate a potential role for probiotics in improving the efficacy of ICI therapy in NSCLC patients.

However, findings in melanoma patients are less conclusive. In a cohort of 158 melanoma patients, probiotic use was not significantly associated with outcomes of ICI therapy (54). In this study, all patients who reported any frequency of probiotic supplement use were designated as probiotic users, and they consumed various types of probiotic formulas and foods. Instead, patients who consumed sufficient dietary fiber (≥ 20 g/day) had longer PFS than those consuming less (not reached vs. 13 months) (n = 128). Notably, the most significant benefit was observed in patients with adequate dietary fiber intake and no probiotic use. Preclinical models of melanoma immunotherapy confirmed these results, highlighting the harmful effects of probiotics and the beneficial effects of dietary fiber on immunotherapy outcomes (54). The discrepancies in the effectiveness of probiotics across different studies may stem from variations in probiotic strains, administration duration, dosage, or cancer types, emphasizing the need for further research.

### 5.2 Intervention studies

In addition to observational studies, numerous clinical trials are underway to assess whether gut microbiota-targeted interventions can improve immunotherapy efficacy in cancer patients. Below, we summarize results from published clinical trials to date (**Table 4**).

**Table 4:** Gut microbiota-based interventions to improve responses to ICI therapy.

Cancer	Intervention	Immunotherapy	Sample size	Design	Phase	Response	NCT	Countries	Reference
Melanoma	FMT	anti-PD-1	10	Single-arm, open-label	1	1 CR, 2 PR	NCT03353402	Israel	Baruch et al., 2021 (55)
Melanoma	FMT	anti-PD-1	15	Single-arm, open-label	2	1 CR, 2 PR, 3 SD	NCT03341143	USA	Davar et al., 2021 (56)
Melanoma	FMT	anti-PD-1	20	Single-arm, open-label	1	4 CR, 9 PR	NCT03772899	Canada	Routy et al., 2023 (57)
GC, ESCC, HCC	FMT	anti-PD-1	13	Single-arm, open-label	N/A	1 PR, 5 SD	NCT04264975	Republic of Korea	Kim et al., 2024 (58)
RCC	CBM588 (*Clostridium butyricum*)	anti-PD-1+CTLA-4	Treatment: 20 Control: 10	Two-arm, randomized, open-label	1	Treatment: 11 (58%) PR Control: 2 (20%) PR	NCT03829111	USA	Dizman et al., 2022 (59)
RCC	CBM588 (*Clostridium butyricum*)	anti-VEGFR-TKI+PD-1	Treatment: 20 Control: 10	Two-arm, randomized, open-label	1	Treatment: 14 (74%) PR Control: 2 (20%) PR	NCT05122546	USA	Ebrahimi et al., 2024 (60)
Melanoma	SER-401 (an orally delivered *Firmicutes* enriched spore formulation)	anti-PD-1	Treatment: 8 Control: 6	Two-arm, randomized, placebo-controlled, blinded	1b	Treatment: 1 (12.5%) CR, 1 (12.5%) PR Control: 4 (66.7%) PR	NCT03817125	USA	Glitza et al., 2024 (61)

GC, gastric cancer; ESCC, esophageal squamous cell carcinoma; HCC, hepatocellular carcinoma; RCC, renal cell carcinoma; VEGFR, vascular endothelial growth factor receptor; FMT, fecal microbiota transplantation; CBM588, *Clostridium butyricum* MIYAIRI 588 strain; CR, complete response; PR, partial response; SD, stable disease.

Baruch et al. conducted a clinical trial (NCT03353402) investigating FMT in 10 patients with immunotherapy-refractory melanoma. FMT improved the response to anti-PD-1 immunotherapy in three patients. Interestingly, all responding patients received FMT from the same donor, whose gut microbiome had a high relative abundance of *B. adolescentis* (55). Another trial by Davar et al. (NCT03341143) in 15 patients with refractory melanoma found that six patients showed improved clinical responses to anti-PD-1 therapy after FMT. Responders demonstrated a gut microbiome shift toward the donor’s profile, increased CD8+ T cell activation, and decreased interleukin (IL)-8-expressing myeloid cells (56). FMT combined with ICI also showed promising results in a trial with 20 previously untreated patients with advanced melanoma (NCT03772899), where 65% of patients (13/20) achieved a response. In responders, donor–recipient gut microbiome similarity increased over time (57). A recent trial (NCT04264975) examined the combined therapeutic effect of anti-PD-1 and FMT on 13 non-responders to anti-PD-(L)1 therapy with advanced solid tumors, including GC, HCC, or esophageal squamous cell carcinoma (58). Clinical benefits were observed, with an ORR of 7.7% and a disease control rate of 46.2%. In the study, the authors isolated *Prevotella merdae* Immunoactis from a responder to FMT and found that it promoted T-cell activity and suppressed tumor growth (58). These findings suggest FMT could be a viable strategy to enhance the efficacy of solid cancer immunotherapy.

In the previous section, we discussed several retrospective studies associating the use of CBM588 before and/or during ICI therapy with better therapeutic outcomes in patients with NSCLC (51–53). In line with the results of the retrospective studies, two phase 1 clinical trials using CBM588 have shown encouraging results (59, 60). In treatment-naïve patients with metastatic RCC (mRCC), the addition of CBM588 to nivolumab–ipilimumab therapy significantly improved PFS compared to controls (12.7 months vs. 2.5 months) (NCT03829111) (59). Another trial added CBM588 to cabozantinib (a vascular endothelial growth factor receptor inhibitor) and nivolumab therapy in treatment-naïve mRCC patients, leading to a significantly higher ORR (74% vs. 20%) (NCT05122546) (60). In these two trials, the patients in the experimental arm received CBM588 at a dose of 80 mg orally twice daily in addition to ICI therapy, where each 40 mg sachet consisted of approximately 2.0 × 10^8^ colony-forming units of *C. butyricum* and pharmaceutical excipients such as corn starch, calcium carbonate, and lactose. These studies indicate that CBM588 may enhance ICI efficacy in mRCC, though larger trials are necessary to validate these results.

Recently, Glitza et al. reported a phase 1 trial (NCT03817125) involving patients with ICI-naïve metastatic melanoma treated with SER-401 (61). SER-401 is a proprietary formulation of bacterial spores from Ruminococcaceae and other spore-forming microbes derived from a stool sample provided by a healthy donor with a gut microbial profile similar to that of immunotherapy responders (19). In this trial, the patients in the SER-401 arm received vancomycin preconditioning followed by SER-401 and then nivolumab with SER-401 maintenance, while the patients in the placebo arm received placebo preconditioning followed by placebo microbiota modulation and then nivolumab with placebo maintenance. This trial was initiated before studies connected antibiotic use before ICI treatment with worse responses to therapy (26, 61), and vancomycin preconditioning was used to prime the gut microbiota for the expansion of SER-401 bacteria in the intestine. In terms of safety and response, the combination of SER-401 and nivolumab was safe, but the ORR in the SER-401 group was lower than in the placebo group (25% vs. 66.7%). In the SER-401 group, the relative abundance of Ruminococcaceae was significantly reduced after vancomycin preconditioning, and it was restored to near-baseline levels after the administration of SER-401. However, no further increase occurred, indicating the suboptimal colonization of target species. Vancomycin preconditioning led to a decrease in the enrichment of ICI response-associated pathways (e.g., butyrate biosynthesis pathway) and an increase in that of ICI resistance-associated pathways in the gut microbiome of the SER-401 group. In addition, antibiotic treatments increased the proportion of peripheral blood mononuclear cell-derived Tregs and circulating serum proteins, which are involved in cellular stress/death, T-cell receptor (TCR) signaling, and Toll-like receptor (TLR) signaling, indicating increased systemic inflammation (61). Several meta-analyses have reported that a high systemic immune inflammatory index or neutrophil-to-lymphocyte ratio (NLR > 3.0), as quantitative measurements of systemic inflammation in the human body, are associated with worse clinical outcomes of OS and PFS in patients with cancer who receive ICI treatment (62, 63). Additionally, in melanoma patients treated with anti-PD-1, the gut microbiome of non-responders was enriched with Gram-negative bacteria and lipopolysaccharide synthesis-related microbial genes, and the host genes encoding pro-inflammatory cytokines were more highly expressed in stool specimens from non-responders than in those from responders. Also, patients with a high NLR had shorter OS and PFS than those with a low NLR (44). Vancomycin, which was used for antibiotic preconditioning in the SER-401 trial, is not effective against Gram-negative bacteria (64). Thus, vancomycin preconditioning might have hindered efforts to determine whether SER-401 could enhance the efficacy of ICI therapy, as it can affect the outcomes of ICI therapy by inducing changes in the intestinal environment and systemic immune responses. This underscores the importance of carefully considering preconditioning regimens when designing clinical trials.

## 6 Mechanisms by which the gut microbiota influences ICI responses

Applications using the gut microbiota to enhance ICI therapy outcomes are of considerable clinical interest. However, a comprehensive understanding of the molecular mechanisms underlying this effect is crucial for deepening our knowledge and developing more effective strategies to harness the gut microbiota for ICI therapy. In this context, we will briefly explore the molecular mechanisms by which the gut microbiota may impact the efficacy of ICI therapy, with a particular focus on the bacterial cell components and metabolites.

### 6.1 Bacterial cell components

Mechanistic studies of *Enterococcus* and *Bacteroides* have revealed the involvement of microbe-associated molecular patterns in enhancing the efficacy of ICI therapy. For instance, immunotherapy-active *Enterococcus* strains secrete the peptidoglycan hydrolase secreted antigen A, which can generate muramyl dipeptide (MDP) peptidoglycan fragments by breaking down the bacterial cell wall. These MDP fragments activate nucleotide-binding oligomerization domain 2-dependent signaling pathways, induce the expression of pro-inflammatory genes, and promote cytotoxic T-lymphocyte (CTL) activation within the tumor microenvironment, consequently resulting in the enhancement of anti-PD-L1 antitumor efficacy (65). Vétizou et al. showed that the administration of *B. fragilis*-purified polysaccharide in antibiotic-treated mice could improve the efficacy of CTLA-4 blockade by inducing IL-12-dependent Th1 immune responses. However, a polysaccharide purified from another *Bacteroides* species, *B. distasonis*, showed lower efficacy in reducing tumor size than that from *B. fragilis* (17). These results indicate that bacterial cell components may improve the efficacy of ICI therapy in a species- or strain-specific manner.

Another type of microbial component, cyclic diadenylate AMP (cdAMP), may also modulate ICI responses. *Akkermansia*-derived cdAMP can act as stimulator of interferon genes (STING) agonist in the tumor microenvironment and improve the antitumor response by inducing IFN-I production and activating NK cells (66). A combination of anti-PD-1 and a synthetic STING agonist, MSA-2, led to significantly inhibited tumor growth compared to that with anti-PD-1 monotherapy in mouse tumor models by inducing CD8+ T-cell infiltration in tumors (67). These findings provide some insight into the mechanism by which *Akkermansia* may promote ICI response.

### 6.2 SCFAs

SCFAs (butyrate, acetate, and propionate) are major metabolites produced by the gut microbiota and play various roles in regulating host physiology. In particular, SCFAs heavily modulate the host immune system by regulating intestinal barrier integrity as well as innate immune cells (e.g., neutrophils, macrophages, and DCs) and adaptive immune cells (e.g., Tregs, Th cells, and CTLs) (68). Preclinical and clinical studies have also reported that SCFAs are involved in ICI responses. In patients with solid tumors, responders to anti-PD-1 inhibitor therapy had higher levels of serum and fecal SCFAs than non-responders (69, 70). In melanoma-bearing mouse models, butyrate supplementation with anti-PD-1 therapy significantly reduced tumor volume and increased the percentage of IFN-γ+ and tumor necrosis factor (TNF)-α+ cells among tumor infiltrating CD4+ and CD8+ T cells. In addition, *in vitro* experiments showed that butyrate promoted the production of antitumor-related cytokines such as IFN-γ, TNF-α, and IL-2 in CD8+ T cells through the TCR-dependent signaling pathway (70). Among gut microbiota, microbes belonging to the *Clostridium* cluster, such as *Faecalibacterium*, *Ruminococcus*, and *Roseburia*, are major producers of butyrate (71). Kang et al. found that *R. intestinalis* abundance was significantly higher in responders to anti-PD-1 therapy than in non-responders among patients with NSCLC. Moreover, in a mouse model of CRC with the microsatellite stable phenotype, *R. intestinalis* or sodium butyrate improved anti-PD-1 efficacy by enhancing cytotoxic CD8+ T-cell function via TLR5-dependent NF-κB pathway activation (72). However, there are conflicting findings regarding the effect of SCFAs on ICI efficacy. Coutzac et al. reported that in patients with metastatic melanoma, low baseline serum butyrate levels were associated with better outcomes of ICI therapy. In mice, sodium butyrate inhibited the anti-CTLA-4-induced maturation of DCs and accumulation of tumor-specific T cells and memory T cells and diminished the antitumor effect of CTLA-4 blockade treatment (73). Therefore, further studies are needed on the role and mechanisms of action of SCFAs in modulating cancer immunity and ICI efficacy for various doses and cancer types.

### 6.3 Inosine

In murine tumor models, *Bifidobacterium pseudolongum* significantly improved the therapeutic efficacy of ICI treatment. Inosine, derived from *B. pseudolongum*, was identified as a key metabolite contributing to this effect. Mechanistically, inosine binds to the adenosine A_2A_ receptor on T cells, which promotes IL12Rβ2 and IFN-γ transcription and Th1-cell activation (74). In addition to *B. pseudolongum*, *A. muciniphila* can produce inosine via inosine–A_2A_ receptor signaling, enhancing the efficacy of ICI treatment (74).

### 6.4 Tryptophan metabolites

Tryptophan metabolites from gut microbes have been studied for their role in immunotherapy responses. Indole-3-carboxylic acid (ICA), produced by *Lactobacillus gallinarum*, inhibits indoleamine 2,3-dioxygenase expression and kynurenine (Kyn) production in tumors. ICA also inhibits Kyn binding to the aryl hydrocarbon receptor in T cells, reducing Treg differentiation and improving anti-PD-1 therapy response in CRC (75). Additionally, ICA from *Lactobacillus johnsonii* has been shown to promote the generation of progenitor-exhausted CD8+ T cells, significantly improving the efficacy of CD8+ T cell-mediated anti-PD-1 immunotherapy across multiple cancer types (76).

### 6.5 Secondary bile acids

Gut microbe-produced secondary bile acids can directly regulate adaptive immune cells. For example, 3-oxolithocholic acid inhibits Th17 cell differentiation by binding to retinoid-related orphan receptor-γt, while isoallolithocholic acid enhances Treg cell differentiation by inducing mitochondrial reactive oxygen species production *in vitro*. These effects were also confirmed in mouse models, indicating that these bile acids may have a detrimental effect on immunotherapy response (77). Another study revealed that deoxycholic acid (DCA) suppresses CD8+ T cell responses by inhibiting Ca^2+^-nuclear factor of activated T cells 2 signaling, promoting tumor growth in mice. Disrupting DCA could mitigate tumor growth (78).

These studies underline the immunological connection between the gut microbiota and cancer, suggesting that specific gut microbes, their components, and metabolites could improve immunotherapy responses. However, the exact mechanisms by which the gut microbiota influences cancer progression and immunotherapy outcomes remain to be fully elucidated.

## 7 Discussion

The reviewed studies make it evident that the gut microbiota plays a significant role in influencing patients’ responses to ICI therapy. While the potential for utilizing the gut microbiome to predict or enhance outcomes of ICI therapy is promising, several challenges must be addressed before it can be widely implemented in clinical practice.

A recurring issue is the lack of consensus regarding gut microbial features associated with responses to ICI therapy. Many studies have identified specific microbes which were enriched in responders and non-responders to ICI treatment in various types of solid cancer, but an individual’s gut microbial compositions are influenced by numerous intrinsic and extrinsic factors such as age, diet, geography, medication, and co-morbidities (79). These factors may results in differences in ICI response-related microbes among the study populations. Therefore, large-scale research projects including diverse populations are needed to identify robust biomarkers applicable to diverse populations. It is also important to collect comprehensive patient metadata such as antibiotic use, co-morbidities, co-medications, and dietary records. These metadata can be used to account for confounding factors in statistical analyses and to identify ICI response-associated features specific to certain subgroups.

Studies investigating gut microbiome-based machine learning models to predict patient responses to ICI therapy have shown promising results. To determine whether a developed model can be applied to other populations, the predictive performance of the model should be evaluated in independent validation cohorts (80). However, it may be inappropriate to use microbiome data produced using different protocols for sample collection, storage, processing, and/or sequencing for validation, as these differences can introduce systemic bias in sequence-based microbiome data (81). Therefore, standardized protocols for microbiome analysis are required in this research area. McCulloch et al. used an empirical Bayesian framework to reduce between-cohort heterogeneity when integrating and analyzing microbiome data from five independent cohorts (44). This method may represent a solution that enables data produced by various protocols to be utilized in the creation and validation of predictive models. Additionally, most predictive models reported to date have been generated using only gut microbiome information. Research is also needed to determine whether adding other known biomarkers for responses to ICI therapy—such as tumor mutational burden and PD-L1 expression—or other omics data can improve the predictive performance of machine learning models.

The gut microbiota holds potential as a modifiable target to enhance the efficacy of ICI therapy. Gut microbes enriched in responders could serve as candidates for LBPs to improve treatment outcomes. Given that strains from the same species can have different functional capabilities (82), high-resolution microbiome studies are necessary to identify specific strains with the greatest potential for boosting the therapeutic efficacy. Additionally, verifying the functional characteristics of candidate microbes through *in vitro* and *in vivo* experiments is critical.

One challenge in gut microbiota interventions is the pre-existing microbial environment. As demonstrated by the SER-401 trial, where the effectiveness of ICI therapy was influenced by antibiotic preconditioning (61), the gut microbial dysbiosis induced by antibiotic treatment before intervention may impact the intervention outcome. Among the four FMT clinical trials mentioned in the previous section, those by Baruch et al. and Kim et al. included the administration of oral antibiotics before FMT treatment for 3 and 5 days, respectively, to remove the native gut microbiota (55, 58). However, the studies by Davar et al. and Routy et al. did not include any antibiotic treatment prior to FMT (56, 57). As these four FMT studies have shown the clinical benefits of FMT combined with ICI therapy regardless of antibiotic preconditioning, the procedure for native microbiota depletion may be omitted in combined treatment using ICIs and gut microbiota manipulation. Further research is needed to determine preconditioning regimens for preparing the gut microbial environment to maximize the effect of the combination therapy. In addition, understanding the relationship between the baseline gut microbial composition and intervention outcomes could help develop microbiome-based stratification strategies for patient treatment.

In addition to FMT and LBPs, ongoing clinical studies are investigating the role of diet and prebiotics in modulating the gut microbiota to enhance the efficacy of ICI therapy. However, our understanding of the mechanisms through which the gut microbiota influences ICI therapy outcomes is still limited. Therefore, large-scale observational and intervention studies, combined with mechanistic investigations, are essential for advancing gut microbiome-based personalized therapeutic strategies applicable in real-world clinical settings.
